# Genetic and epigenetic analysis of putative breast cancer stem cell models

**DOI:** 10.1186/1471-2407-13-358

**Published:** 2013-07-24

**Authors:** Marija Balic, Daniela Schwarzenbacher, Stefanie Stanzer, Ellen Heitzer, Martina Auer, Jochen B Geigl, Richard J Cote, Ram H Datar, Nadia Dandachi

**Affiliations:** 1 Division of Oncology, Department of Internal Medicine, Medical University of Graz, Auenbruggerplatz 15, A-8036 Graz, Austria; 2 Institute of Human Genetics, Medical University of Graz, Graz, Austria; 3 Department of Pathology, University of Miami Miller School of Medicine, Miami, FL, USA

## Abstract

**Background:**

Cancer stem cell model hypothesizes existence of a small proportion of tumor cells capable of sustaining tumor formation, self-renewal and differentiation. In breast cancer, these cells were found to be associated with CD44^+^CD24^-low^ and ALDH^+^ phenotype. Our study was performed to evaluate the suitability of current approaches for breast cancer stem cell analyses to evaluate heterogeneity of breast cancer cells through their extensive genetic and epigenetic characterization.

**Methods:**

Breast cancer cell lines MCF7 and SUM159 were cultured in adherent conditions and as mammospheres. Flow cytometry sorting for CD44, CD24 and ALDH was performed. Sorted and unsorted populations, mammospheres and adherent cell cultures were subjected to DNA profiling by array CGH and methylation profiling by Epitect Methyl qPCR array. Methylation status of selected genes was further evaluated by pyrosequencing. Functional impact of methylation was evaluated by mRNA analysis for selected genes.

**Results:**

Array CGH did not reveal any genomic differences. In contrast, putative breast cancer stem cells showed altered methylation levels of several genes compared to parental tumor cells.

**Conclusions:**

Our results underpin the hypothesis that epigenetic mechanisms seem to play a major role in the regulation of CSCs. However, it is also clear that more efficient methods for CSC enrichment are needed. This work underscores requirement of additional approaches to reveal heterogeneity within breast cancer.

## Background

Breast cancer is the most commonly diagnosed cancer and the leading cause of cancer death in women [1]. Despite combined treatment strategies and advances in treatment, metastatic breast cancer remains currently incurable. One of the possible reasons for therapeutic failure is the existence of tumor cell heterogeneity and presence of cancer stem cells (CSCs) [2]. There are several indicators of intratumoral heterogeneity, including recognized prognostic and predictive markers and candidate biomarkers [3,4]. Among the clinically established biomarkers are estrogen and progesterone receptor, and human epidermal growth factor receptor 2 (Her2-neu) [5]. An emerging cellular candidate biomarker is the presence of breast CSCs [6,7]. Increasing experimental evidence supports the cancer stem cell model [8], which is in favor of only a small proportion of cells with the capability of sustaining tumor formation and growth, self-renewal and differentiation. In breast cancer, CSCs have been identified as CD44^+^CD24^-/low^ or aldehyde dehydrogenase positive (ALDH^+^) [9,10]. Several approaches have been used to enrich and study breast CSCs, including flow cytometry sorting and their capability of forming mammospheres [9,11]. Tumor sphere culture has been shown to represent a surrogate *in vitro* model to study CSCs [12,13]. Identification of distinct properties and molecular biomarkers for breast CSCs may help to identify novel therapeutic targets and thereby allow development of more efficient therapeutic strategies [14].

We aimed to evaluate molecular heterogeneity of breast cancer cell lines with an emphasis on breast CSCs. For unsorted breast cancer cells and flow-sorted putative stem versus non-stem cells, DNA profiles were generated by array comparative genomic hybridization (aCGH) and methylation analyses of selected candidate genes were done by pyrosequencing. Functional impact of methylation was evaluated by mRNA analysis for selected genes.

## Methods

### Breast cancer cell lines

Breast cancer cell lines MCF7 and SUM159 were used for all experiments. SUM159 were obtained from Asterand (Detroit, MI, USA), and MCF7 from American Type Culture Collection (Manassas, VA, USA). Cells were cultured according to suppliers’ recommendations, harvested at 90% confluence and prepared for further analyses.

### Mammosphere culture

The culture of mammospheres (MMO) was performed according to previous publications [11]. Briefly, cells were grown in serum-free Mammary Epithelial Basal medium MEBM (Lonza, Basel, Switzerland), supplemented with 10 ng/mL basic fibroblast growth factor (bFGF), 20 ng/mL epidermal growth factor (EGF), 5 μg/mL insulin (both from Peprotech, New York, USA), and 20 μl/ml B27 supplement (Invitrogen, Leek, Netherlands). After the first passage, the mammospheres were filtered through a 40-μm Nylon Cell Strainer (BD, Falcon) to obtain purer spheres for further culture. The cells were then dissociated with TrypLE (Gibco/Invitrogen, Paisley, Renfrewshire, UK), following incubation at 37°C for 4 min. The cells were washed with two volumes of PBS (phosphate buffer saline) to inactivate the enzyme, resuspended in MEBM containing supplements and seeded for generation of secondary spheres.

### Flow cytometry

For all sorting experiments cells were dissociated with Accutase (PAA Laboratories GmbH, Pasching, Austria) for 5 min at 37°C. When using the Aldefluor protocol [10], dissociated cells were suspended in Aldefluor assay buffer to a concentration of 10^6^ cells/ml. When performing anti-CD44 and anti-CD24 flow-sorting [9], 10^6^ cells were suspended in 100 μl of PBS/2% fetal calf serum (FCS). After 20 min of incubation on ice with blocking buffer consisting of horse serum diluted 1:20 in 6% bovine serum albumin/PBS solution, aliquots of antibodies (CD44 APC and CD24 FITC), each at a dilution of 1:40 in a final concentration of 0.08 μg/ml and 5 μg/ml, respectively, were added and staining was adapted from the previously published protocol [9]. Briefly, after centrifugation the pellet was dissolved in 100 μl PBS/FCS and 2.5 μl of the antibodies (CD24-FITC and CD44-APC) were mixed with the cells and incubated for 20 min on ice. Then, cells were washed with 500 μl PBS/FCS and centrifuged again. Before sorting, cells were resuspended in 100 μl PBS/FCS, filtered and rinsed with 100 μl PBS/FCS. All fluorochrome-labeled monoclonal antibodies were acquired from BD Bioscience and pretitered to determine their optimal dilutions before use. Cells without staining and isotype controls, also from BD Bioscience, were integrated as controls in all experiments.

#### Aldefluor assay

Cells with high ALDH activity in MCF7 and SUM159 cells were isolated using the Aldefluor Kit (StemCell Technologies, Durham, NC, USA) according to the manufacturer’s instructions and as previously published [10,15]. Cells isolated with Aldefluor Kit were used for genetic and epigenetic analysis. Flow cytometry sorting was performed on the Aria fluorescence activating cell sorter (FACS) and acquired data were analyzed using the Diva software (BD Bioscience).

### DNA extraction

Genomic DNA from cultured cells was extracted using the Gentra Puregene Blood Kit (Qiagen) according to the manufacturer's protocol. DNA was dissolved in a final volume of 100 μL buffer and quantified spectrophotometrically using a BioPhotometer (Eppendorf, Hamburg, Germany).

### Evaluation of genomic profiles

#### Whole genome amplification (WGA)

DNA from sorted SUM159 subpopulations (ALDH^+^, ALDH^-^, CD44^+^CD24^-^, CD44^+^ CD24^+^) and manually picked SUM159 and MCF7 mammospheres were amplified using the GenomePlex Single Cell Whole Genome Amplification Kit (Sigma-Aldrich, St. Louis, MO, USA) following the instructions of the manufacturer. To prevent any loss, cells were sorted directly into tubes where cell lysis and WGA were performed. Briefly, the volume was adjusted with water to 9 μl. After cell lysis and Proteinase K digest the DNA was fragmented and libraries were prepared. These products were used as templates for the amplification reaction which was performed in a thermal cycler (95°C for 3 min, 25 cycles of 94°C for 30 seconds and 65°C for 5 min, hold 4°C) by adding 7.5 μL of 10 × Amplification Master Mix, 48.5 μL of nuclease-free water and 5 μL WGA DNA polymerase. Amplified samples were purified using GenElute PCR Clean-up Kit (Sigma-Aldrich, St. Louis, MO, USA) and quantified by measuring absorbance on a NanoDrop Spectrophotometer (Thermo Scientific, MA; USA). The quality of the amplification was evaluated using a multiplex PCR [16].

Samples with significantly lower than the expected average DNA concentration of 250 ng/μl after WGA, or samples that showed only one band in multiplex PCR were excluded from further analyses.

#### Array CGH (aCGH)

Array CGH was carried out using a genome-wide oligonucleotide microarray platform (Human genome CGH 60 K microarray kit, Agilent Technologies, Santa Clara, CA, USA), following instructions of the manufacturer, and employing commercially available male reference DNA (Promega, Madison, WI, USA). Briefly, 500 ng DNA from SUM159 and MCF7 was digested with restriction endonucleases AluI/RsaI at 37°C for two hours, followed by an enzyme inactivating step at 65°C for 20 min. A smear between 2000 and 100 bp on a 1% agarose gel indicated successful digestion. Due to the previous fragmentation during the WGA this step was omitted for amplified samples (e.g. DNA of cultured MMO of both cell lines and sorted SUM159).

Samples were then labeled with the Bioprime array CGH genomic labeling system (Invitrogen, Carlsbad, CA, USA) according to the manufacturer’s instructions. Five hundred ng each of the test DNA and the reference DNA were differentially labeled with dCTP-Cy5 or dCTP-Cy3 (GE Healthcare, Milwaukee, WI, USA). Unincorporated nucleotides were removed using the Amicon KD30 Kit (Millipore, Billerica, MA, USA). The probes were quantified before hybridization by determining the absorbance at 260 nm (DNA), 550 nm (cyanine 3), and 650 nm (cyanine 5) using NanoDrop spectrophotometer and denatured by incubation at 95°C for 2 min followed by cooling to room temperature. After annealing for 30 min at 37°C, array hybridization was carried out at 65°C for 24 hours with about 200 ng probes/array in Agilent HI-RPM hybridization buffer. Slides were scanned using a microarray scanner (#G2505B; Agilent Technologies, Santa Clara, CA, USA), and images processed using Feature Extraction and DNA Workbench 5.0.14 (Agilent Technologies). In addition, data normalization and calculation of ratio values was performed using the Feature Extraction software 9.1 from Agilent Technologies. Evaluation of data was done based on the previously published algorithm in R ( http://www.r-project.org) [17]. The algorithm calculates ratio values with different window sizes that differ significantly from the mean of the ratio profile and are therefore considered as over- or under-represented graphically indicated in a single green or red bar for gained or lost regions, respectively. Furthermore, the algorithm generates a table with all localizations of significant calls, which allows detailed mapping of each copy number variation (CNV).

### Evaluation of epigenetic profiles

#### Epitect methyl qPCR arrays

To identify relevant gene promoter methylation we used EpiTect Methyl qPCR Arrays (SA Biosciences, Qiagen, Hilden, Germany) as a screening method. We analyzed 96 cancer-related genes from four pathways including stem cell transcription factors, homeobox genes, wnt signaling, and epithelial to mesenchymal transition (EMT). Assays were performed according to the manufacturer’s instructions. Results were displayed as percentage of unmethylated and methylated fraction.

#### Pyrosequencing

For methylation analysis by pyrosequencing, one μg of genomic DNA was subjected to bisulfite conversion with the EpiTect Bisulfite Kit (Qiagen) according to the manufacturer's instructions. The purified bisulfite converted samples were eluted in 40 μl volume and stored at −20°C for further analysis.

To quantify the percentage of methylated cytosine in individual CpG sites, bisulfite-converted DNA was analyzed using pyrosequencing (Pyromark® Q24, Qiagen) as previously described [18]. Custom Pyromark® CpG assays were used for following genes: HOXB4, HDAC1, FOXA2, CTBP1, LEF1, SMAD2, DSC2 and HIF1A. For genes HOXD3 and WIF1 assays were designed using the Pyromark Assay Design Software Version 2. Following primers were used: HOXD3-fw-5′-AGTTAAAGGTTATTTTAAAGGTTTAT-3′, HOXD3-Bio-rev-5′-CCTCTTACATCTACCCTATACAATT-3′, HOXD3-Pyro 5′-GGTTATTTTAAAGGTTTATGG-3′ and WIF1-fw-5′-GGGGGAGTTGTGGGGTTTTT-3′, WIF1-Bio-rev-5′-CCCAAAAATCTCTAAATACCCTTCTC-3′, WIF1-Pyro-5′-TGTGGGGTTTTTTAGGGGG-3′.

### Real-time quantitative PCR (qPCR)

Total RNA was extracted from parental adherent cells, MMO or sorted cells using TRIzol Reagent (Invitrogen, Carlsbad, CA, USA) according to the manufacture’s recommendation. RNA was quantified and assessed for purity by UV spectrophotometry. One microgram of total RNA was reverse transcribed using the QuantiTect Reverse Transcription Kit (Qiagen) according to the manufacturer’s instructions.

qPCR was performed using LightCycler 480 (Roche). Reactions were performed in a total volume of 20 μl, comprising 1x SYBR Green I Master Mix (Roche), 20 ng cDNA and 25 μM of each primer (final concentration). All qPCR reactions were performed in duplicate and quantification cycle values were averaged. Gene expression was calculated by the comparative Ct method [19] and relative mRNA expression was presented as fold-differences over that in the parental monolayer cells. Hydroxymethylbilane synthase (HMBS) expression was used as internal control.

Following RT-qPCR primers were chosen from the public database ( http://www.rtprimerdb.org) [20,21], gene (RTPrimerDB-ID): HOXD3 (4603), HIF1A (7974), RGS2 (7770), CTBP1 (4471), LEF1 (8496), HDAC1 (442) and HMBS (4). The program Primer3 (NCBI, Primer-BLAST, http://www.ncbi.nlm.nih.gov/tools/primer-blast) was used to design WIF1 primer sequences (WIF1-fw-5′-GGAAAATGTATTTGCCCTCCA-3′ and WIF1-rv-5′-CCAATGCATTTACCTCCATTTC-3′).

### Statistical analysis

Quantitative data are represented as the mean ± SD. For comparison of means, we used Student’s *t*-test and ANOVA with Tukey multiple comparison as a post hoc analysis. Statistical significance was defined as *p* < 0.05. GraphPad Prism software version 6 (GraphPad Software, La Jolla, California, USA) was used for statistical analysis.

## Results

### Putative stem cell phenotypes in breast cancer cell lines

**Figure 1:**
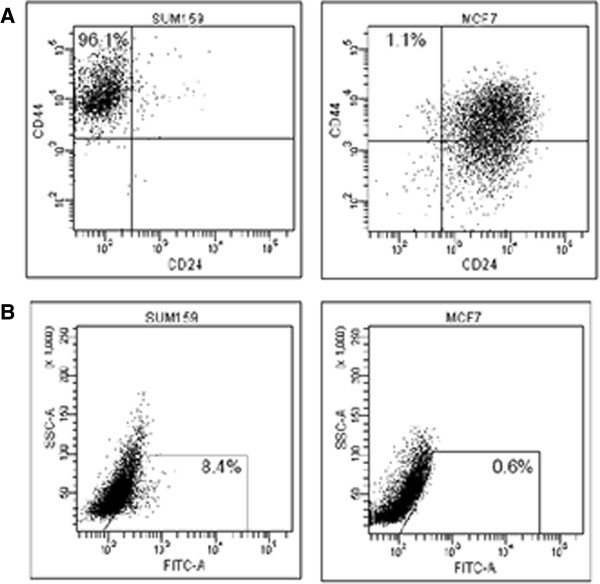
**Expression of putative stem cell markers in breast cancer cell lines MCF7 and SUM159. (A)** FACS analysis measuring CD44 and CD24 expression in SUM159 (left) and MCF7 (right) breast cancer cell line. The percentages reflect the population of putative breast cancer stem cells defined as CD44^+^CD24^-/low^. **(B)** Aldefluor analysis measuring ALDH expression in SUM159 (left) and MCF7 (right) breast cancer cell line. The percentages reflect the Aldefluor positive population of the cells.

Flow cytometry was used to assess the expression of putative stem cell markers in MCF7 and SUM159 cells, including CD44, CD24, and ALDH. As shown in Figure 1A, MCF7 cells had a low amount of CD44^+^CD24^-/low^ cells (1.1%), whereas SUM159 cells showed a high proportion of CD44^+^CD24^-/low^ cells (96.1%). In addition, the ALDH activity was evaluated. Figure 1B illustrates that MCF7 cells showed low ALDH positivity (0.6%), in contrast to SUM159 cells where we detected an increased ALDH activity (8.4%).

These results indicate that SUM159 cells have an enriched stem cell phenotype.

### Mammosphere formation assay

**Figure 2:**
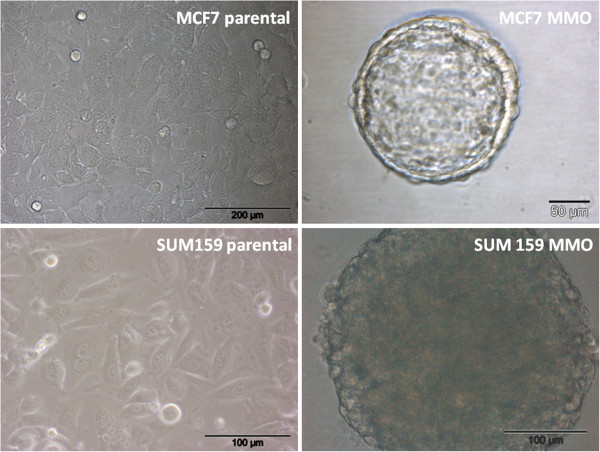
**Representative images from parental MCF7 and SUM159 cells and corresponding MMO.** SUM159 formed significantly larger spheres within half of the time as compared to MCF7.

MCF7 and SUM159 were both able to generate MMO in non-adherent conditions. For generation of MMO in first passage, MCF7 remained in non-adherent conditions for 10 days. After filtration and dissociation of spheres, another 10 days were needed for generation of secondary spheres. SUM159 cells were able to form spheres after 5 days. Analogous to the MCF7 MMO culture, secondary spheres were generated, which required 5 days. Parental cultures and corresponding representative spheres are depicted in Figure 2. As indicated in the figure and previously suggested [22], SUM159 were capable of forming significantly larger spheres within half of the time. Single spheres and pools of spheres were manually picked and subjected to further profiling analyses.

### Genomic profiles of putative breast CSCs

We employed aCGH to analyze 10 manually picked spheres each from MMO cultures of SUM159 and MCF7 and compared them with cells grown in adherent culture.

**Figure 3:**
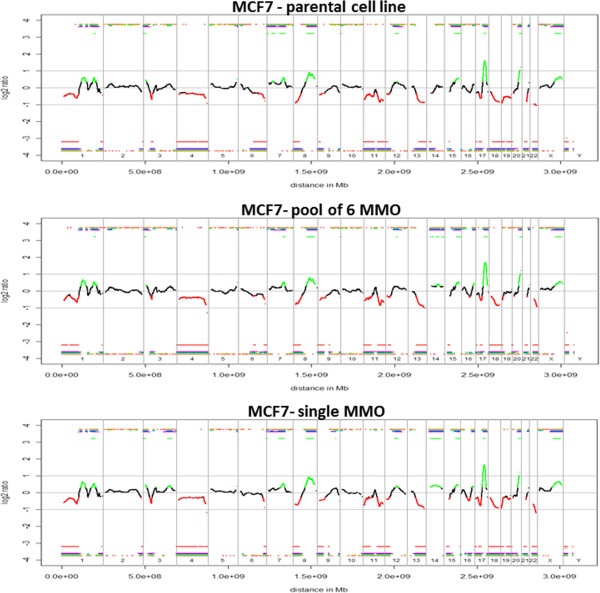
**Array CGH profiles of MCF7 parental cell line, a pool of 6 MMO and a single MMO.** No significant copy number differences of MCF7 between genomic DNA of the parental cell line and the corresponding MMO were observed. Black parts in the profile represent balanced regions, lost regions appear in red and gained regions in green.

**Figure 4:**
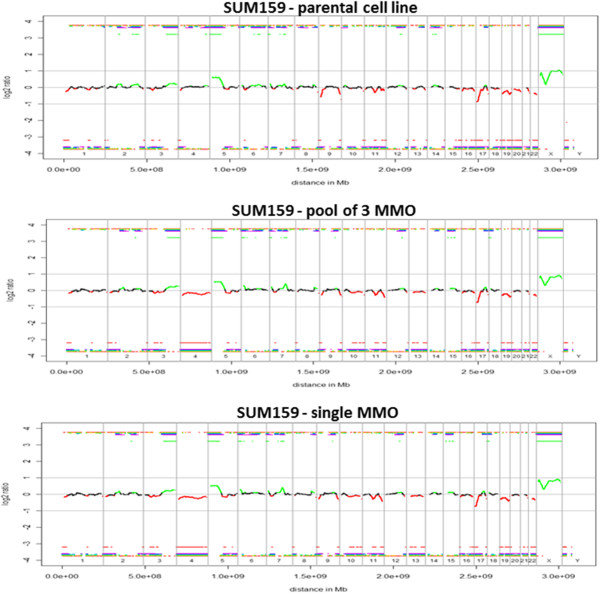
**Array CGH profiles of SUM159 parental cell line, a pool of 3 MMO and a single MMO.** No significant copy number differences of SUM159 between genomic DNA of the parental cell line and the corresponding MMO were observed. Black parts in the profile represent balanced regions, lost regions appear in red and gained regions in green.

From both SUM159 and MCF7 two spheres were excluded from further analyses due to either low DNA concentrations following WGA or incomplete target amplification in the multiplex PCR. All other spheres showed 4 bands after PCR, with an average yield of 10 μg and 12 μg for SUM159 and MCF7, respectively. We observed no significant copy number differences for MCF7 between genomic DNA of the parental cell line and the corresponding MMO. All samples showed the same genomic aberrations including high-level gains at 8q, 15q, 17q and 20q, and losses at 1p, 8p, 11q, 11q, 13q, 18q and 22q. Most regions of chromosome 4 were underrepresented (Figure 3). These changes are in line with previously published copy number profiles for MCF7 [23,24]. For SUM159 we obtained similar results. We observed high-level amplifications of 3q and 5p, losses at 17p and 21q, and a complete loss of chromosomes 4, 19 and 22 in all samples (Figure 4).

**Figure 5:**
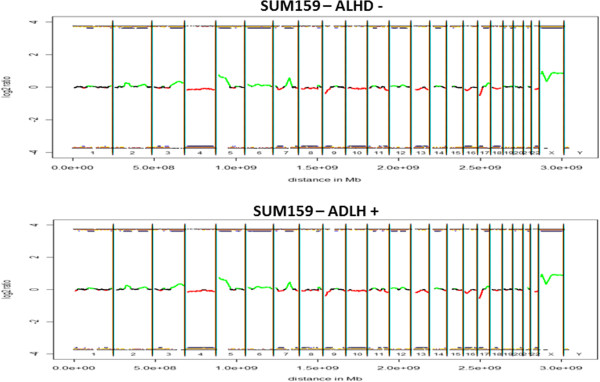
**Array CGH profiles after separation of SUM159 ALHD**^**+ **^**and ALDH**^**- **^**subpopulation.** No significant copy number differences of SUM159 between ALDH^+^ and ALDH^-^ subpopulation were observed. Black parts in the profile represent balanced regions, lost regions appear in red and gained regions in green.

**Figure 6:**
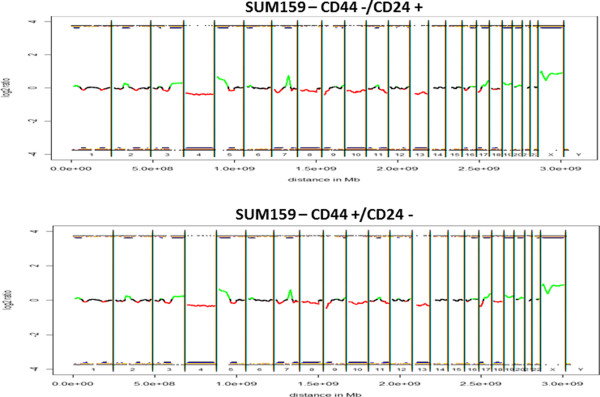
**Array CGH profiles after separation of SUM159 between CD44**^**+**^**CD24**^**- **^**and CD44**^**-**^**CD24**^**+ **^**subpopulation.** No significant copy number differences of SUM159 between CD44^+^CD24^-^ and CD44^-^CD24^+^ subpopulation were observed. Black parts in the profile represent balanced regions, lost regions appear in red and gained regions in green.

Since MMO displayed no significant copy number variation compared to the parental cell lines, we further wanted to determine whether different cell subpopulations of SUM159 exhibit distinct differences in their genomic aberration profiles. After flow sorting, four subpopulations (ALDH^+^, ALDH^-^, CD44^+^CD24^-^, CD44^+^CD24^+^) were subjected to WGA followed by aCGH. Again, we did not detect any difference in the copy number profile even after separation of cell populations (ALDH^+^ and ALDH^–^ cells are shown in Figure 5 and CD44^+^CD24^-^ cells vs. CD44 ^–^CD24^+^ in Figure 6).

**Figure 7:**
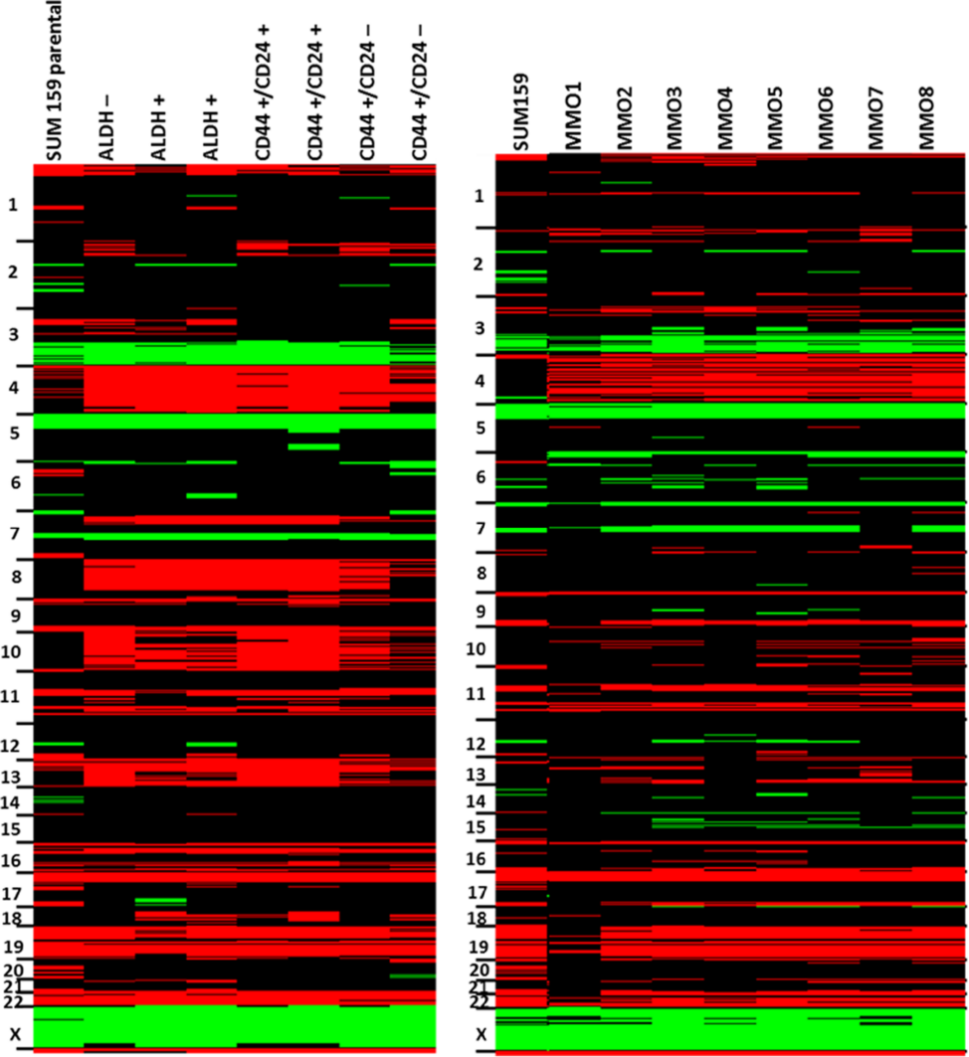
**Heat maps of CGH-profiles from SUM159 parental cell line compared to sorted subpopulations and to single MMO.** We used male reference DNA in all experiments and therefore female plasma DNA samples have a relative over-representation of the X chromosome and an under- representation of the Y-chromosome (black: balanced; red: under-represented; green: over-represented).

**Figure 8:**
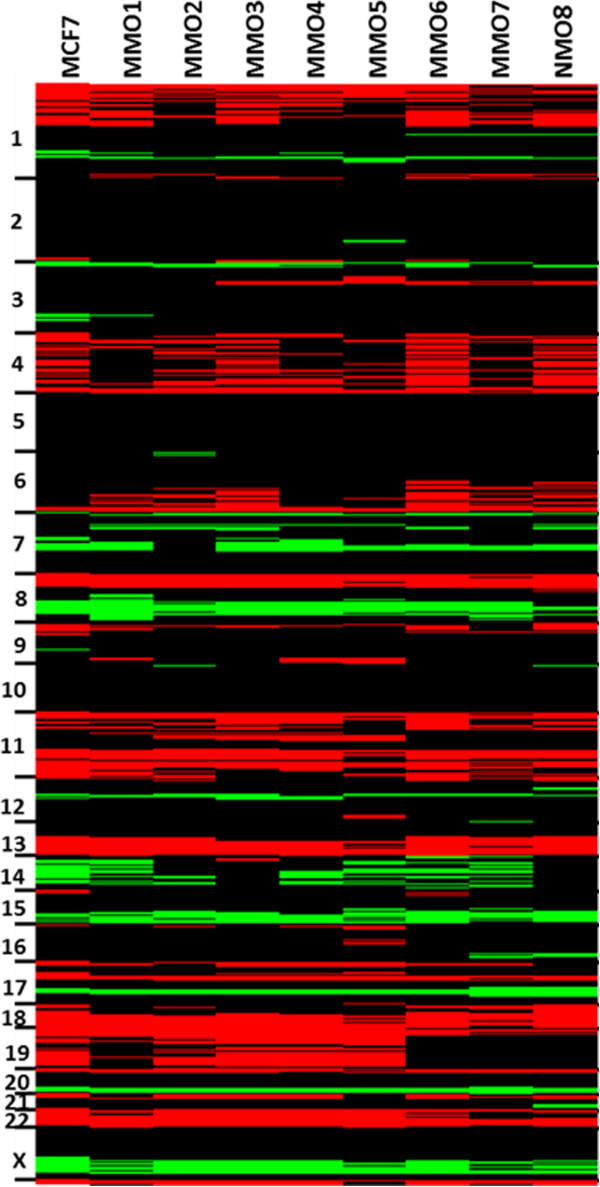
**Heat maps of CGH-profiles from MCF7parental cell line compared to single MMO.** We used male reference DNA in all experiments and therefore female plasma DNA samples have a relative over-representation of the X chromosome and an under- representation of the Y-chromosome (black: balanced; red: under-represented; green: over-represented).

This data is summarized in heat maps of aCGH profiles for SUM159 (Figure 7) and MCF7 (Figure 8), respectively. Minor differences in CNV that mainly include single, non-adjacent oligonucleotides most likely represent artifacts introduced during the amplification process. Nevertheless, amplification artifacts with the single cell amplification, which may result in under- (e.g. allele drop out) or over-representations (e.g. preferential amplifications) are probably rare as we previously reported [17].

### Methylation analysis of putative breast CSCs

**Figure 9:**
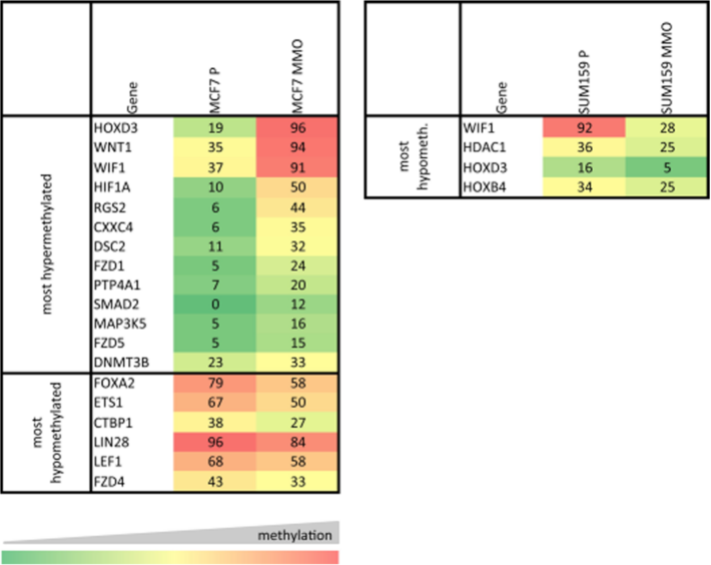
**Differential promoter methylation profile of candidate genes in parental breast cancer cells (MCF7 P and SUM159 P) and putative breast CSC (MMO).** Methylation status of 96 genes was analyzed using the Epitect Methyl qPCR Arrays (Qiagen). Methylation level is expressed in a 0-100% scale, with higher methylation shown in red and lower methylation shown in green. For MCF7, we identified a set of 19 genes differentially methylated in mammospheres compared to parental cells. 13 of these genes were hypermethylated and six genes were hypomethylated in mammospheres. For SUM159, we found only four differentially methylated genes and all these genes were hypomethylated in mammospheres.

Based on the assumption that heterogeneity of the cells may be driven by epigenetic changes, we screened promoter methylation of 96 candidate genes in parental breast cancer cells and putative breast CSC using Epitect Methyl qPCR Arrays (results are summarized in Figure 9). We selected a panel of candidate genes showing differential methylation for further methylation analysis using bisulfite pyrosequencing. These genes included HOXD3, WIF1, HIF1A, RGS2, DSC2, SMAD2, FOXA2, CTBP1 and LEF1 for MCF7 and WIF1, HDAC1, HOXD3 and HOXB4 for SUM159. Two biological replicates of each condition were included in the pyrosequencing analysis. Again, parental cells and MMO were analyzed. In addition, the sorted subpopulations (ALDH^+^, ALDH^-^, CD44^+^CD24^-^, CD44^+^CD24^+^) from SUM159 cells were also analyzed. Sorted MCF7 cells could not be analyzed since the percentage of cells with putative breast cancer stem cell phenotype was too low.

**Figure 10:**
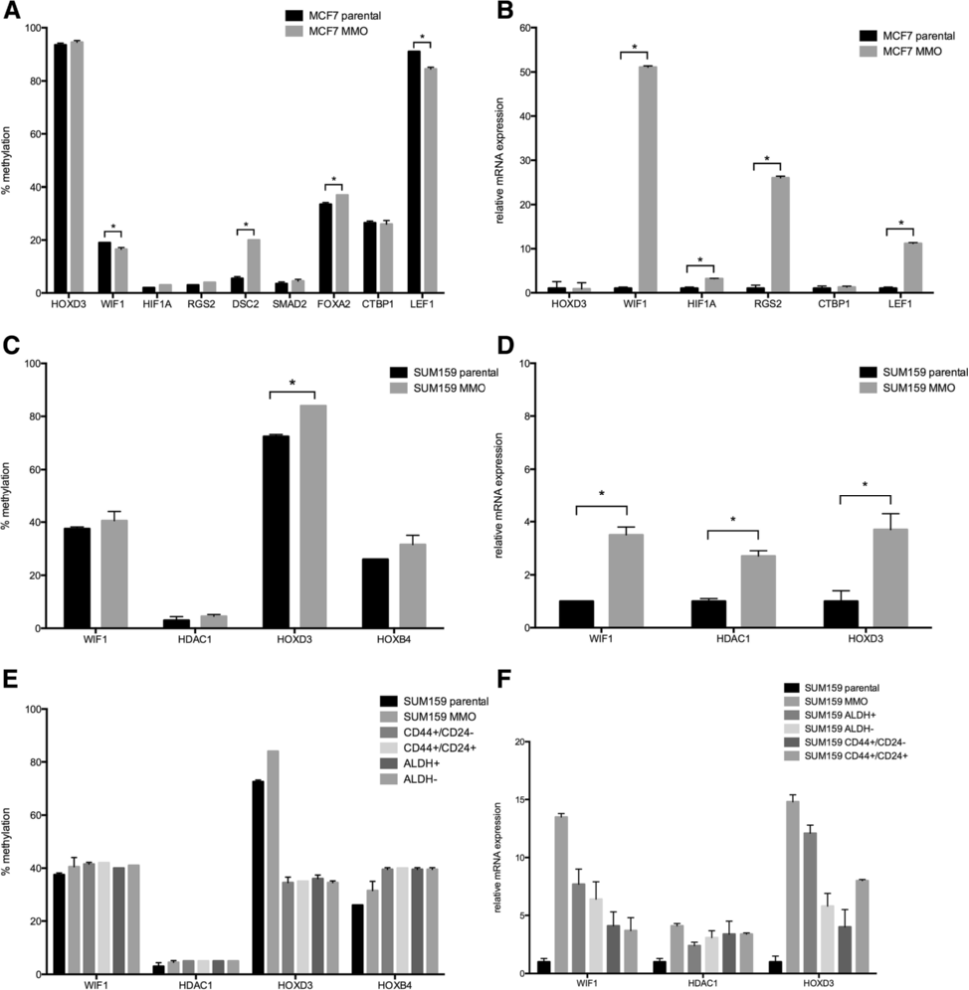
**Association between DNA promoter methylation and mRNA expression of selected candidate genes in parental MCF7 and SUM159 cells and their corresponding CSC phenotype.** DNA methylation was assessed by pyrosequencing and mRNA expression by qRT-PCR. Bars represent average and SD of two biological replicates. *p < 0.05. **(A)** DNA promoter methylation and **(B)** mRNA expression levels in parental and MMO MCF7 cells. **(C)** DNA promoter methylation and **(D)** mRNA expression levels in parental and MMO SUM159 cells. **(E)** DNA promoter methylation and **(F)** mRNA expression levels in sorted subpopulations of SUM159 cells.

In MCF7, four of 9 genes showed significantly different methylation levels between parental and MMO cells. These genes included WIF1, DSC2, FOXA2 and LEF1 (Figure 10A). Compared to MMO, WIF1 and LEF1 showed lower methylation levels, while DSC2 and FOXA2 showed higher methylation levels. To test whether methylation of candidate gene promoters affects the expression of corresponding genes, we analyzed mRNA expression levels in parental and MMO cells. WIF1, HIF1A, RGS2 and LEF1 showed a significantly higher expression in MMO compared to parental MCF7 cells (Figure 10B). WIF1 and LEF1 showed an inverse relationship between methylation level and mRNA expression. HOXD3 and CTBP1 mRNA levels were similar in MMO and parental MCF7 cells. This is in line with the methylation results, which were also similar in both conditions.

Generally, Epitect Methyl qPCR Arrays revealed less detectable methylation differences in SUM159 cell line. Applying bisulfite pyrosequencing, four selected candidate genes showed higher methylation levels between MMO and parental cells, with HOXD3 being the only gene with significantly higher levels (Figure 10C). Regarding gene expression, WIF1, HDAC1 and HOXD3 mRNA levels were significantly higher in SUM159 MMO cells than in parental cells (Figure 10D). Hence, there was no inverse correlation between HOXD3 methylation and gene expression.

To test whether culturing tumor cells in non-adherent condition could affect methylation of the genes analyzed, we also subjected sorted subpopulations of SUM159 cells (ALDH^+^, ALDH^-^, CD44^+^CD24^-^, CD44^+^CD24^+^) to gene methylation analysis. WIF1 and HDAC1 showed similar levels of gene methylations in all sorted subpopulations and MMO cells, but lower levels for parental SUM159 cells. In contrast, HOXD3 was similarly methylated at lower levels in all sorted populations, but higher methylation levels were found in MMO and parental cells. HOXB4, on the other hand, was similarly higher methylated in sorted populations, but less methylated in MMO and parental cells. These results are summarized in Figure 10E. mRNA expression levels of WIF1, HDAC1 and HOXD3 were different in distinct sorted populations compared to MMO and parental SUM159 cells (Figure 10F).

## Discussion

This is the first study to analyze both genetic and epigenetic alterations in putative breast cancer stem cell models derived from established breast cancer cell lines. At the genomic level we did not find any copy number variations between putative breast cancer stem cells (i.e. mammospheres or sorted subpopulations) and the corresponding parental tumor cells. In contrast, putative breast cancer stem cells showed altered methylation levels of several genes compared to parental tumor cells.

Definition and analysis of CSCs remains a challenge. Among the approaches to enrich and study the cancer stem cell biology are *in vitro* cultures and flow cytometry sorting based on defined putative phenotypes of CSCs for different diseases [9,10,25,26]. We focused our analyses on evaluation of genetic and epigenetic profiles in an attempt to associate the heterogeneity with the presence of putative CSCs within breast cancer cell lines. Parental cell lines and MMO originating from the parental cell line and distinct populations defined by putative breast cancer stem cell phenotypes were compared.

Two biologically distinct breast cancer cell lines were used: MCF7 represents luminal breast epithelial tumor cells, and SUM159 represents triple negative breast epithelial tumor cells. The phenotypes we used to enrich for breast CSCs were established earlier by Al Hajj *et al.*[9] and Ginestier *et al.*[10], and are commonly used to address breast CSCs. Ability of these two cell lines to form MMO was previously demonstrated by us and others [15,22,27]. The proportions of putative breast CSC subpopulations we observed in both cell lines were comparable with published results [22,28,29].

This is the first comparison of aCGH profiles between parental cells and MMO for both cell lines, and indicates that there is no difference in genomic aberrations across the analyzed samples. One explanation for the similar genotype could be the fact that CSCs give rise to all progenitor cells and therefore both share the same genetic profile. However, studies suggest that cancers may contain multiple CSC clones, arising through clonal evolution in CSCs [8]. Such CSC clones are more likely to be found in primary tumors rather than in cell lines. In addition, although CSCs are enriched in these models, not all cells within the mammosphere population represent cells with stem cell properties. As a result, the extent of dilution of the CSC signature, both genetically and epigenetically, is unknown.

Genomic profile of MCF7 cells was comparable with results published for adherent cell cultures [23,24]. We did not find matching data for SUM159. As expected, owing to their very distinct biology, MCF7 and SUM159 had substantially different genomic profiles.

Proposed plasticity and dynamic property of cancer cells, often associated with the ability to undergo EMT and reverse and to switch stem cell markers off and back on, suggest an underlying epigenetic mechanism [30-32]. These properties determine metastatic potential of tumors [33], and may be the reason why most disseminated tumor cells in bone marrow have the putative breast CSC phenotype, as we have shown previously [34]. Epigenetic regulation of common CSC genes has recently been demonstrated in triple negative breast cancer [35]. Furthermore, in a recent paper, Park et al. have demonstrated distinct methylation pattern across defined intrinsic molecular breast cancer subtypes and their correlation with described breast CSC markers in archival primary breast cancer tissues [36]. In concordance to our findings in breast cancer cell lines, primary cancer tissues of triple negative breast cancer had higher frequency of ALDH^+^ and CD44^+^CD24^-^ cells. Therefore, our further analyses focused on analyzing methylation profiles of putative breast CSCs *in vitro*. Genes screened by methylation arrays were chosen according to the potentially important molecular pathways involved in stem cell biology, including stem cell transcription factors, homeobox genes, wnt signaling and EMT. Selected differentially methylated genes were further evaluated by bisulfite pyrosequencing.

Bisulfite pyrosequencing revealed differences in methylation levels between parental and MMO cells and also between sorted populations and MMO cells. Differences in sorted subpopulations may reflect not only the heterogeneity in CSCs, but also different ability to enrich for CSC. As already stated, enrichment factor for CSCs is unknown for both approaches. In case of the SUM159 cell line, the majority of cells were associated with CD44^+^CD24^-^ CSC phenotype. These cells are more aggressive, basal like, with mesenchymal characteristics, and with most of the cells being similar, detection of differentially expressed markers is less likely.

Several groups have used a similar approach in attempt to associate intratumoral heterogeneity with the presence of CSCs. Clearly, the main goal of such an approach is to understand the biological mechanism and finally identify biomarkers of CSCs to design better treatment strategies. One of the first studies was published by Hernandez-Vargas *et al.*[37], revealing JAK-STAT pathway activation in putative breast CSCs defined as CD44^+^CD24^-^ MCF7 cells. Hypomethylation of genes in JAK- STAT pathway was demonstrated further in MMO. The authors provided not only a potential definition of biomarkers associated with CSCs, but also an evidence of underlying epigenetic regulation of CSC properties. In a similar analysis in hepatocellular carcinoma, researchers identified epigenetic regulation of wnt signaling as a potential biomarker of early detection and therapeutic targeting [38]. Epigenetic mechanisms and the role of miRNA regulation of CSCs have also been demonstrated [39,40] providing a new insight into biology and potential definition of novel therapeutic targets. All these analyses are promising and require further evaluation.

Finally, our mRNA analyses suggest that although some genes showed an inverse correlation between methylation and gene expression, other mechanisms than DNA promoter methylation may be responsible for regulating gene expression. In our study, methylation seemed to play a role only in the regulation of WIF1 and LEF1 in MCF7. One reason for the lacking correlation might be heterogeneous promoter methylation and that at least in part our methylation assay did not detect the regulating region. Also, further mechanisms might be involved in epigenetic silencing of proteins, including histone modifications, long noncoding RNAs and miRNAs [41]. These mechanisms are incompletely understood and are emerging study topics. However, their evaluation was out of the scope of the present study.

Our results, along with others, clearly underpin the hypothesis that epigenetic mechanisms play a major role in the regulation of CSCs, however, more efficient methods for CSC enrichment are needed. Other mechanisms of epigenetic regulation have also to be taken into account. Moreover, these analyses should be extended to patient samples, in order to validate results, and possibly find higher heterogeneity than possible and present in cell lines.

## Conclusions

In conclusion, stem cell hypothesis has to be further challenged; we need additional markers and more efficient approaches and models in order to better define the biology of stem cells and identify biomarkers for early diagnosis and treatment with higher precision.

## Abbreviations

CSCs: Cancer stem cells; ALDH: Aldehyde dehydrogenase; aCGH: Array comparative genomic hybridization; MMO: Mammospheres; WGA: Whole genome amplification

## Competing interests

The authors declare that they have no competing interests.

## Authors’ contributions

MB and ND conceived and supervised the study. DS, MA, EH and SS performed the experiments. ND, MB, EH and JG analyzed and interpreted the data. MB, ND, RD and RC drafted and revised the manuscript. Rd and RC provided technical support. All authors read and approved the final manuscript.

## Pre-publication history

The pre-publication history for this paper can be accessed here:

http://www.biomedcentral.com/1471-2407/13/358/prepub
